# Highly Selective Enzymatic Recovery of Building Blocks from Wool-Cotton-Polyester Textile Waste Blends

**DOI:** 10.3390/polym10101107

**Published:** 2018-10-07

**Authors:** Felice Quartinello, Sara Vecchiato, Simone Weinberger, Klemens Kremenser, Lukas Skopek, Alessandro Pellis, Georg M. Guebitz

**Affiliations:** 1 Department of Agrobiotechnology IFA-Tulln, University of Natural Resources and Life Sciences Vienna, Institute of Environmental Biotechnology, Konrad Lorenz Strasse 20, 3430 Tulln an der Donau, Austria; felice.quartinello@boku.ac.at (F.Q.); guebitz@boku.ac.at (G.M.G.); 2 Austrian Centre of Industrial Biotechnology, Division Polymers & Enzymes, Konrad Lorenz Strasse 20, 3430 Tulln an der Donau, Austria; sara.vecchiato@acib.at (S.V.); simone.weinberger@boku.ac.at (S.W.); klemens.kremser@boku.ac.at (K.K.); lukas.skopek@boku.ac.at (L.S.); 3 Department of Chemistry, University of York, Green Chemistry Centre of Excellence, Heslington Yo105DD, UK

**Keywords:** textile recycling, sustainable processes, protease, amino acids, cellulases, bioethanol, poly(ethylene terephthalate), circular economy

## Abstract

In Europe, most of the discarded and un-wearable textiles are incinerated or landfilled. In this study, we present an enzyme-based strategy for the recovery of valuable building blocks from mixed textile waste and blends as a circular economy concept. Therefore, model and real textile waste were sequentially incubated with (1) protease for the extraction of amino acids from wool components (95% efficiency) and (2) cellulases for the recovery of glucose from cotton and rayon constituents (85% efficiency). The purity of the remaining poly(ethylene terephthalate) (PET) unaltered by the enzymatic treatments was assessed *via* Fourier-transformed infrared spectroscopy. Amino acids recovered from wool were characterized via elementary and molecular size analysis, while the glucose resulting from the cotton hydrolysis was successfully converted into ethanol by fermentation with *Saccharomyces cerevisiae*. This work demonstrated that the step-wise application of enzymes can be used for the recovery of pure building blocks (glucose) and their further reuse in fermentative processes.

## 1. Introduction

In the last decades the production of textiles, especially for clothing, is exponentially increasing, mostly due to globalization [1,2,3]. This intensive production is responsible for the decreases in prices leading the consumers to consider clothes as disposable materials. From the estimation provided by Hollins (based on an extrapolation of data provided by nine textile sorters in EU countries), 80,000 tons of textile waste are generated per year [4]. As is the case for other solid wastes, textile waste comprises three types: (1) pre-consumer (obtained from fiber processing and manufacturing); (2) post-consumer (all the textiles that the consumers discard either because they are damaged or gone out of fashion) and (3) post-industrial, which is generated from commercial and industrial applications [5]. The end-life of such material is landfilling that, together with soil pollution, represent a global warming challenge due to the production of gases [6,7]. For example, only 18% of this kind of waste in the last years was used for energy recovery [8]. Furthermore, considering material composition, the discarded textiles still contains valuable polymers that could be reused. Resource depletion, climate change and rising consumer awareness are providing challenges to governments and industrial systems to find new solutions towards improved and environmentally friendly waste-management systems and treatments. Most of the collected items could be exported separated in wearable and un-wearable textiles. Therefore, such un-wearable fabrics can be used for recycling lowering the environmental impact of clothing waste, consequently matching the most recent environmental legislation [9]. Textile waste recycling could save around 4.2 trillion gallons of water, 17 million tons of CO_2_ and 7.5 million cubic yards of landfill space [10].

Textiles are composed of both natural and synthetic materials. Natural fibers include cellulose-based materials (cotton, viscose, linen or hemp) or to a lesser extent protein-based materials like wool and silk [11]. On the other hand, polyester is the most common synthetic polymer and is also used blended with the above-listed materials [1,12,13]. Blended materials, like cotton-polyester, wool-polyester and cotton-wool, allow the tuning of the properties of the fabrics such as wettability or softness and in parallel represent a reduction in the production costs. However, blends represent a challenge in terms of separation and recycling; due to the interconnection of the fibers, a separation method (mechanical or chemical) as a pretreatment is required. Similarly, mixed textile waste is difficult to separate.

**Scheme 1:**
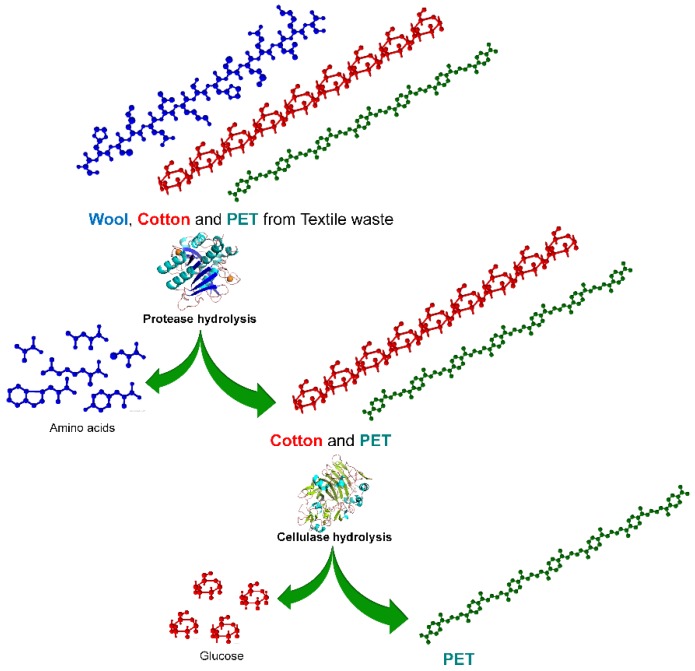
Graphic representation of the highly selective enzymatic recovery of building blocks from wool-cotton-polyester textile blends waste materials. The process involves a first hydrolysis step of wool using proteases followed by a second enzymatic hydrolysis of cellulose using cellulases and it’s nicely finished by the poly(ethylene terephthalate) (PET) hydrolysis using cutinases.

Enzymes, due to their high specificity, would allow step-wise recovery of the components of blended materials under environmentally friendly conditions [14,15]. Although enzymatic hydrolysis of individual components has been demonstrated by us and other groups [16,17,18,19], the potential of enzymes for stepwise recovery of building blocks from blends has not been demonstrated so far. Hence, in this study, step-wise enzymatic hydrolysis of cellulose/wool/polyester blends was investigated. In this work, a stepwise enzymatic process which can specifically separate the three different polymers present in textile waste was developed. Moreover, the enzymatic treatment achieved the preservation of the functional properties of the relative hydrolyzed products, which are suitable as secondary value-added products for different industrial applications (Scheme 1).

## 2. Materials and Methods

### 2.1. Chemicals, Substrates and Enzymes

The different samples of cellulose, wool, polyester and their blends were provided from the SOEX group (Hamburg, Germany). All the other chemicals and solvents were purchased from Sigma-Aldrich (Vienna, Austria) at reagent grade and used without any purification if not specified. The used enzymes, Cellic CTec3® and Savinase 12T® were kindly provided by Novozymes (Copenhagen, Denmark), and used without prior purification steps.

### 2.2. Enzymatic Hydrolysis

**Figure 1:**
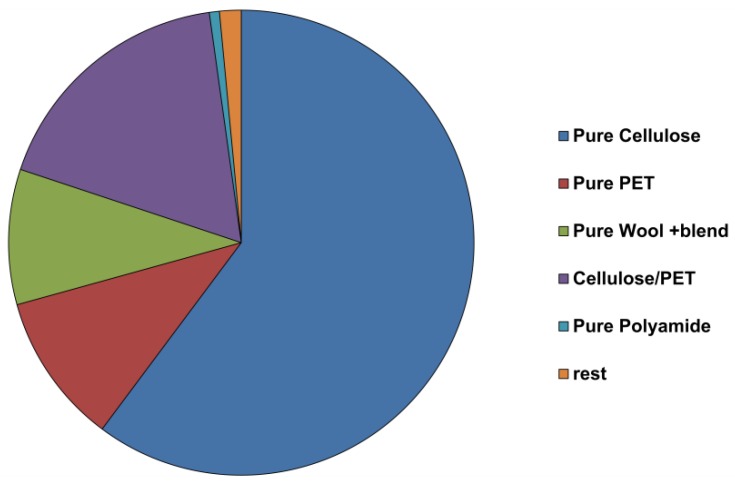
Composition of the mixed textile waste used in the present study. Data were kindly provided by the SOEX Group (Hamburg, Germany).

The mixed samples consisted of pure cellulosic material (61%), pure polyester (11%), cellulose/PET blends (18%), wool and wool blends (2%), polyamide and other materials (8%) (Figure 1).

Additionally, various mixtures with a known percentage of pure cellulose, polyester and wool textiles were prepared (Table 1).

All the samples were ground to a size of 1 mm, in order to increase the available area for the enzymatic treatment and improve the mass transfer [20]. The artificial blends were washed in mQ-H_2_O heated up at boiling temperature for 30 min and subsequently dried at 105 °C for 6 hours. The same treatment was performed for the real samples from textile waste. 1 g of each sample was weighted and incubated with 75 mL of 50 mM Tris-HCl buffer pH 9 (containing 1 g·L^−1^ of SDS and 6 g·L^−1^ of sodium bisulfite) containing 8 U mL^−1^ (ratio with solid matter) of protease for two days at 50 °C and stirred at 400 rpm on a magnetic stirrer [21]. Afterwards, samples were vacuum-filtered with PES 0.2 µM filter (Millipore, Austria), added into 75 mL of mQ-H_2_O, and stirred for 30 min at 400 rpm. Later, samples were again filtered and dried at 105 °C for 6 hours.

Subsequently, 75 mL of 50 mM sodium citric buffer pH 4.8 with 2750 U mL^−1^ of cellulase cocktail were incubated for 5 days at 400 rpm and 50 °C [22]. After the hydrolysis of the cellulose moieties, each sample was filtered and dried as described above. Before and after each step (hydrolysis and washing steps), the samples were weighted. All measurements were performed in triplicates. The yield of the hydrolysis treatment was calculated using equation 1.

(1) $$Yield=\frac{W_{a}-W_{b}}{W_{a}}*100$$

where *W*_a_ = weight of the initial (or previous) step; *W*_b_ = weight after the step of interest.

### 2.3. Protein Hydrolysate Characterization

#### 2.3.1. Protease Activity and Thermal Stability Assay

The activity of the protease was determined at different pH levels of the buffer (8, 9 and 10), with and without SDS and Sodium bisulfite using the azocasein-assay [22,23]. In order to start enzymatic hydrolysis, 75 μL of enzyme solution (1% *w*/*w*) were mixed with 125 μL of a 2% azocasein solution. Pure buffer without the enzyme was used for the determination of the blank. After 30 min of incubation at 37 °C and shaking at 300 rpm, the reaction was stopped by adding 600 μL TCA (10%). Afterwards the solution was incubated for 15 min at 25 °C. The samples were then centrifuged for 5 min at 20 °C and 13,000 rpm in order to precipitate the not hydrolyzed casein. Then 600 μL of the supernatant was mixed with 700 μL of NaOH (1 M) subsequently 220 μL of the mixture was transferred into a 96 well plate and the absorbance was measured at 440 nm using an Infinite M 200 plate reader (Perkin Elmer, Traiskirchen, Austria). The determination of the absorbance was performed in triplicates. The thermal stability of the enzyme was determined using the same azocasein assay as described above. Briefly, the enzyme solution was incubated at 50 °C and 300 rpm. The aliquots were taken at 0, 3, 6, 24 and 48 h after the beginning of the degradation process and the remaining enzymatic activity was evaluated.

#### 2.3.2. Chemical Analysis

Total carbon (TC), total inorganic carbon (TIC) and total nitrogen (TN) were determined on a Shimadzu System composed of a TOC-VCPH total organic carbon analyzer and a TNM-1 nitrogen measurement unit, equipped with ASI-V auto sampler unit. The measurement range of TC was 10 to 1000 mg·L^−1^, of TIC 10 to 100 mg·L^−1^ and TN 20 to 200 mg·L^−1^. If necessary, samples were diluted accordingly with ultrapure water. Determination limits were determined according to DIN32645 as 4.45 mg·L^−1^ for TC, 5 mg·L^−1^ for IC and 4.39 mg·L^−1^ for TN. TOC was calculated as the difference of TC and TIC.

#### 2.3.3. Molecular Weight Distribution of Keratin Hydrolysis

The keratin extraction during the enzyme treatment was followed via SDS-PAGE analysis. A volume of 20 μL of each sample obtained from the different textile waste hydrolysates were mixed with 20 μL of Laemmli buffer and heated for 5 min. Afterwards, 12 μL of solution was transferred into a precast polyacrylamide gel (4%–15%), using 5 μL of protein marker IV (10–170 KDa, 10 bands). Moreover, 1 g of untreated pure wool was stirred with a 10 mL solution of 50 mM Tris-HCl pH 8.5, containing 2-mercaptoethanol (1.5 M), urea (8 M) and SDS (0.25 M). Then 20 μL of this solution was run in SDS-PAGE as previously described [24].

#### 2.3.4. Amino Acid Determination

In order to determine the amino groups present in the supernatant after protease treatment the ninhydrin assay was performed. Therefore, a ninhydrin reagent, consisting of 75 mL of dimethylsulfoxide (DMSO), 300 mg hydrindantin, 2 g of ninhydrin and 25 mL of 4 M sodium acetate (pH 5.2) was used. Standard samples (0−200 µM) were prepared from a 10 mM stock solution of glycine in mQ-H_2_O. A volume of 100 µL of sample (or standard) was incubated with 75 µL of ninhydrin reagent and incubated for 30 min at 80 °C. Thereafter, samples were cooled down and 100 µL of stabilizing solution (50% of ethanol in water) were added. The absorbance was determined at 570 nm using an Infinite 200 Pro spectrophotometer (Tecan, Switzerland).

### 2.4. Cellulose Hydrolysis

#### 2.4.1. Total Cellulose Assay

The filter paper assay (FPA) was used as recommended by IUPAC [25]. Rolled filter paper (7.5 × 75 mm^2^, around 50 mg each) was submerged in a glass tube with 1 mL 50 mM sodium citrate buffer pH 4.8. Therefore, 100 µL of enzyme (diluted 1:1000) was added to the substrate. The reaction was stopped at different time points (0, 5, 10, 20, 40, 60 min) by adding 500 µL of 1 M NaOH. Specifically, for the first time point at 0 min NaOH was added before the enzyme and considered that reaction as blank. Afterwards, 3,5-dinitrosalicylic acid (DNS) reagent was added and then the sample boiled for 5 min, followed by the addition of 1 mL of mQ-H_2_O [26]. 200 µL of each sample were transferred in a 96 well-plate and the absorbance was measured at 540 nm. All measurements were conducted in triplicates.

#### 2.4.2. Quantification of Glucose *via* HPLC

Carrez precipitation was performed with the supernatant obtained from hydrolyzed cellulosic materials to separate the soluble sugars from the proteins. Briefly, to 960 µL of supernatant was added 20 µL of 2% K_4_[Fe(CN)_6_]·3H_2_O and 20 µL of 2% ZnSO_4_·7H_2_O solutions. Samples were centrifuged at 14.000 rpm at 4 °C for 15 min and filtered through 0.2 µm nylon filter into HPLC vials for measurements. Agilent technologies 1260 Infinity II HPLC, with Transgenomic IC SEP-ION-300 coupled with refractive index detector was performed with H_2_SO_4_ with a flow rate of 0.325 mL·min^−1^ at 45 °C.

#### 2.4.3. Bio-Ethanol Production

After the enzymatic hydrolysis, the supernatant was ultra-filtered through a VivaFlow membrane (5000 MWCO) (Sartorius, Germany), in order to remove impurities. Afterwards, the glucose was passed through a 0.1 µm sterile vacuum filter (EMD, Millipore, Austria). The amount of glucose after the filtration was determined as described above via HPLC and stored at 4 °C until further use.

The glucose released from enzymatic hydrolysis of cellulose was used for ethanol production from *Saccharomyces cerevisiae*. An overnight pre-culture was incubated at 28 °C and 150 rpm in Yeast Extract–Peptone–Dextrose Medium (YPD) media (1% yeast extract, 2% peptone, 2% d-glucose) [19]. Then, the pre-culture as collected by centrifugation washed 3 times and re-suspended in 10 mL of mQ-H_2_O. The yeast was re-inoculated to an optical density (OD600) of 0.1. In this case, the culture media was prepared containing YPD for the control and in the end YPGc (1% extract, 2% peptone and 2% glucose obtained from the textile degradation). The growth of *S. cerevisiae* was monitored by measuring the OD600 after 0, 3, 6, 18, 24 hours. During the same time points, different samples were collected for quantification of glucose and ethanol via HPLC as described before.

### 2.5. Poly(Ethylene Terephthalate) Characterization

After the step-wise enzymatic “extraction” of protein- and cellulose-based materials FT-IR spectra (ATR accessory, Perkin Elmer, Traiskirchen, Austria) of the residual materials (20 mg) were recorded. Poly(ethylene terephthalate) (PET) fabric from GoodFellow was used as standard in order to determine the purity of the PET obtained after the different enzymatic processes. A second blank with ground pure cotton was measured in order to discriminate the signals from the cellulose material from PET peaks. A total of 25 scans for each sample were taken with a resolution of 2 cm^−1^ and normalized before any data processing. The bands were assigned as follows: 3300 cm^−1^ (–OH groups from cellulosic fiber) 870 cm^−1^ (C–H out of plane bending), 1720 cm^−1^ ν(C=O), 1590 cm^−1^ ν(C=C), 1250 cm^−1^ ν(C–O–C).

Enzymatic hydrolysis of residual PET was performed using 5 µM of *Humicola insolens* cutinase (HiC) (Novozyme, Denmark). Therefore, weight loss of PET samples after the enzymatic treatment was quantified as described for protein- and cellulose-based materials.

## 3. Results and Discussion

### 3.1. Step-Wise Enzymatic Extraction of Textile Building Blocks

**Figure 2:**
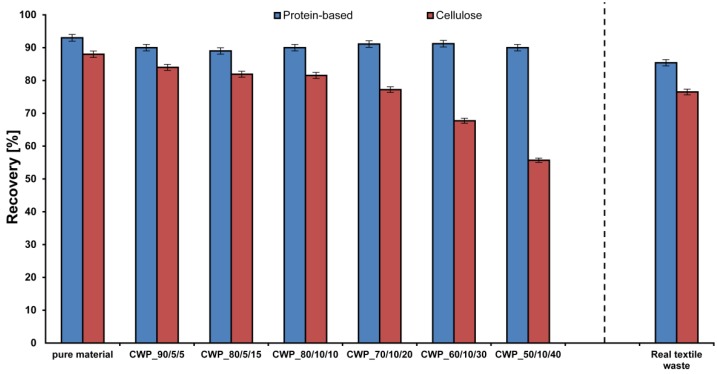
Recovery of protein and cellulose fibers building blocks after sequential treatment of textile waste and model mixtures with proteases and cellulases, respectively depending on cellulose (C), wool (W) and polyester (P) content. The data are given in (%) related to weight loss from the starting material. All experiments were performed in triplicates and plotted ± the standard deviation.

To assess the impact of the material composition on enzymatic hydrolysis, protease and cellulase activities and incubation times were chosen in a way to reach about 90% conversion based on preliminary experiments with pure materials. In the first step, building-blocks of protein-based textile waste components, namely wool, were recovered by protease treatment. About 90% of the protein content of both pure wool and model mixtures was recovered in the solution after protease treatment. For the real textile waste sample, a slightly lower recovery (85%) was measured (Figure 2). Furthermore, no glucose (or oligosaccharides) was detected after protease treatment, indicating the specificity of the protease. In the second step, the recovery of cellulose building blocks (i.e. glucose) from cotton and man-made cellulose fibers was investigated. Higher yields were seen for those samples with higher cellulose content (Figure 2, Samples CWP_90/5/5 and CWP_80/10/10), which decreased to 50% when the polyester increased to 40% (Figure 2, Sample CWP_50/10/40). However, almost 80% of the cellulose fiber building blocks were recovered from real textile waste after cellulase treatment. The washing steps, performed between the enzymatic processes, did not significantly improve yields, i.e., only a slight increase below 1% was seen (see ESI, Figure S1). Regarding the recovery from real textile waste, the yield was 5–10% lower when compared to the model mixture with similar cellulose content, probably due to the intimate connections of the fibers present in the artificial blends.

Wool is a protein fiber 95% of which is composed of the weight of keratin which consists of 18 amino acids with a relatively high proportion of cysteine (10%–15%) [27,28]. Keratin has a high number of disulfide bonds which are responsible for a high stability to the wool protein structure. The cortex consists of shaped cortical cells of lower-sulfur content intermediate filaments (IFP) with an average of 40–65 KDa. These proteins are embedded in a matrix of β-keratin sheets which comprise keratin-associated proteins (KAP). The KAPs contain two non-filamentous proteins rich in cysteine (11–26 KDa) and protein with high glycine, serine and tyrosine content (6–9 KDa). Savinase 12T® is secreted by the alkalophilic bacterium *Bacillus lentus* and is a representative of the subtilisin enzymes with high enzymatic activity in the pH range 8–12 [29].

The activity and the thermal stability of the enzyme were positively influenced by the presence of SDS as a surfactant, due to better dissolution of the enzyme from the granulated powder. Furthermore, sodium bisulfite (which is needed for breaking disulfide bonds in wool) was not affecting the enzyme catalysis (see ESI, Figure S2). Protease activity was slightly higher at pH 9 and pH 10 when compared to pH 8, but the enzyme was no longer stable at pH 10 (see ESI, Figure S3).

**Figure 3:**
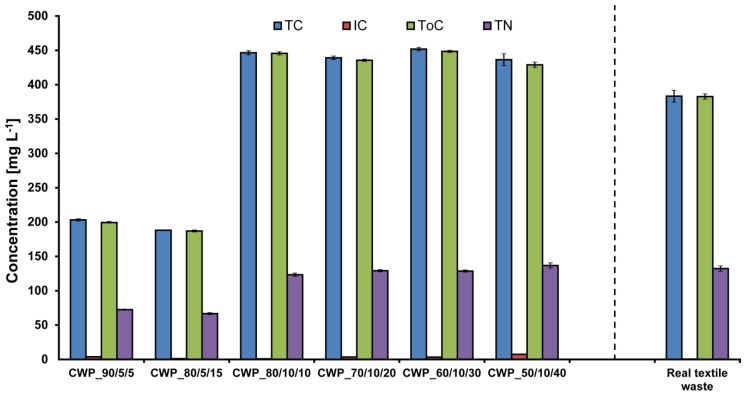
Determination of total carbon (TC), inorganic carbon (IC), total organic carbon (ToC) and total nitrogen of textile fiber sample hydrolysates after the protease treatment. Blank values from TC, IC, ToC and total nitrogen (TN) were subtracted.

The enzymatic solubilization of wool was also quantified based on total organic carbon and the total nitrogen analysis in the solution after removal of the solids. The amounts released correlated well with the initial wool content and the weight loss measured (Figure 3). The carbon/nitrogen ratio was 3:1 in agreement with published values [24] for wool hydrolysates applied for germination application [30,31] or as a phenolic compound replacement [32,33,34]. In the hydrolysate of real textile waste, we observed a similar C/N ratio as it is possible to observe from Figure 3.

Peptides released upon protease treatment had a molecular weight lower than 10 KDa according to SDS-Page analysis (see ESI, Figure S4). Furthermore, amino groups of the peptides/amino acids in the hydrolysates were quantified by using the ninhydrin assay. In this case, the corresponding amount of these moieties is also correlated by the initial amount of protein-based material, showing the same trend as well the ToC and TN (see ESI, Figure S5).

### 3.2. Cellulose-Based Material Hydrolysis and Ethanol Production

The cellulase preparation used for the hydrolysis of cellulose fibers present in textile waste comprised a complete cellulose degrading enzyme system including endoglucanases, cellobiohydrolases, polysaccharide monooxygenases and beta-glucosidases allowing synergistic hydrolysis yielding glucose [35]. Glucose is a perfect carbon source for the biotechnological production of platform chemicals and biofuels. Moreover, when recovered from waste streams there is no ethic concerns related to competition to food production. However, impurities potentially contained in textile waste might inhibit the growth of microorganisms. Hence, production of bioethanol of recovered glucose was exemplary investigated in this study and used as carbon source for *S. cerevisiae* in comparison to commercial glucose.

**Figure 4:**
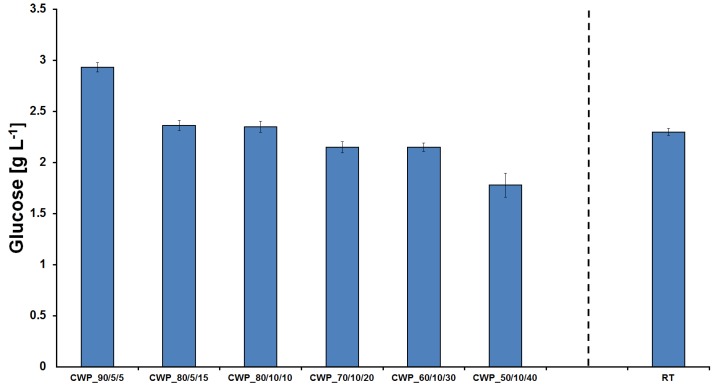
HPLC-RI measurements of recovered glucose from enzymatic degradation of cellulose material depending on cellulose (C), wool (W) and polyester (P) content. RT= real textile waste sample.

The amount of glucose released after hydrolysis of the textile waste samples by cellulases depended on the initial cellulose content. The highest amount was obtained (2.9 g/L) in Sample CWP_90/5/5 (Figure 4). Inversely, samples with lower amounts of such material have shown slightly lower amounts of released sugar (Figure 4). This can be explained by the presence of higher oligosaccharides as previously reported by Vecchiato et al. [19].

**Figure 5:**
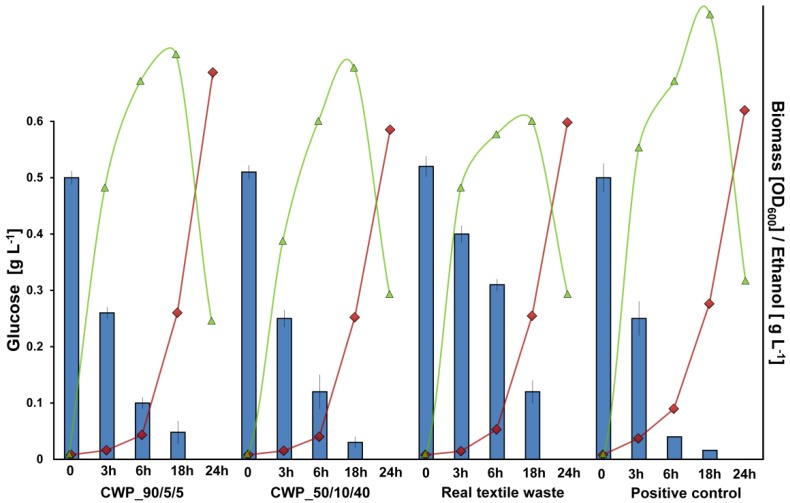
Fermentation of *S. cerevisiae* with the enzymatically recovered glucose. Time points collected after time 0, 3, 6 and 24 hours of incubation of yeast. The concentration of glucose (blue bars) and ethanol (green line) were quantified via HPLC analysis (experiments performed in triplicate). Biomass (red line) was quantified via spectrophotometric measurement of OD600.

Generally, the data reported in Figure 5 indicate that the recycled glucose either from artificial blends (samples CWP_90/5/5 and CWP 50/10/40) and real textile waste (RT) was easily used as the carbon source by the yeast. The amount of sugar drastically decreased after 6 hours and after 24 h there was no more sugar available. After 6 h of fermentation the maximum production of ethanol of around 0.3 g·L^−1^ was observed. Afterwards, the amount of ethanol decreased since the yeast started using it as carbon source due to the glucose lack. Glucose recovered from real textile waste as a carbon source showed an intermediary trend, the yeast growth was marginally affected by the growth with artificial blends. In comparison with the highest value of produced ethanol (0.3 g·L^−1^) after 6 h the incubation with real textile waste produced 0.2 g L^−1^. The real textile waste sample produced 0.2 g·L^−1^ in comparison with the highest value of produced ethanol (0.3 g·L^−1^) from an artificial blend. Certainly, the amount of ethanol produced is lower than at an industrial scale [36] but the fermentation with yeast was performed in a small scale just as the proof of concept of the use of recovered glucose from enzymatic recycling processes. Moreover, it could, indeed, be suitable for ethanol production provided that the process is further optimized.

### 3.3. Characterization of the Recovered Poly(Ethylene Terephthalate) (PET)

**Figure 6:**
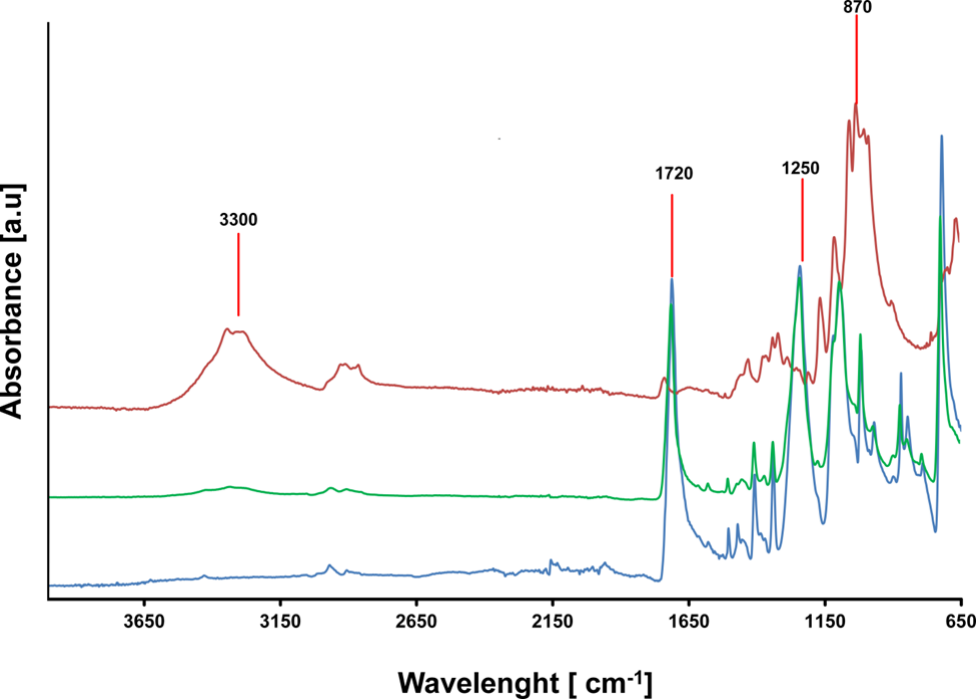
FT-IR real textile waste sample. Pure PET (blue line), pure cotton (red line), real textile waste sample (green line). All the spectra were normalized in the area 2500–2200 cm^−1^.

The high durability and crystallinity of PET is a major concern for the environment. On the other hand, its separation from other fibers can avoid their release into the environment [37] and allow recycling which is otherwise impossible with, e.g., cellulose impurities. As already defined, the two enzymatic steps have the aim to separate the polyester from the two initial natural fibers. FT-IR analysis (Figure 6) demonstrated that, in the samples CWP_90/5/5-CWP_80/10/10, the polymer obtained after the enzymatic treatments was 96% pure, due to the absence of the cellulose peaks, as shown in the blank with pure cotton. On the other hand, the presence of the peaks in the 3300 and 870 cm^−1^ area in samples CWP_60/10/30 and CWP_50/10/40 indicated the traces of cellulose (see ESI, Figure S6). According to the yield, when a higher content of PET (more than 30%) is present, the cellulose removal by the enzyme cocktail is less efficient. Regarding the spectra of the real textile waste sample, after the enzymatic treatments small signals from cellulose material were also present around 870 cm^−1^ with the purity of the recovered PET that was around 92%. However, in general, most of the natural fibers in blended textiles were successfully removed with recovered polyesters with purities greater than 90% in all cases.

Finally, as an alternative to recycling of PET as a polymer, in another enzymatic step the building block could potentially be recovered. Incubation of the residual PET with the cutinase from *Humicola insolens* demonstrated a partial degradation of PET, still not sufficient for complete recycling (see ESI, Figure S7) but efficient enough in order to obtain functionalized materials similarly to what previously reported for other polymers belonging to the class of polyesters such as poly(lactic acid) [38,39]. As shown in our previous work, a complete degradation of PET is possible using a synergistic combination of neutral chemical and enzymatic hydrolysis [40].

## 4. Conclusions

Enzymatic treatment has proven to be an environmentally friendly alternative tool towards the reduction of textile waste. The enzymatic hydrolysis of wool- and cellulose- based fibers led to yields of approximately 95% and 85%, respectively. The purity of the resulting poly(ethylene terephthalate) was comparable to the pure PET as demonstrated by FT-IR measurements, allowing recycling.

Furthermore, in line with circular economy concepts, the recovered building blocks from wool and cellulose fiber components in blended textiles can be reused. The amino acids and oligopeptides (with molecular weight lower than 10 KDa) obtained from wool degradation can be used as a replacement for carbon and nitrogen sources for germination or phenolic-related compounds for resins according to previous reports. On the other hand, in this paper recovered glucose (around 0.62 g·L^−1^) was successfully used as carbon source for yeast fermentation to produce ethanol (0.3 g·L^−1^). In summary, such a step-wise enzymatic process is especially attractive for the recycling of blended materials which are otherwise rather difficult to recycle with other technologies.

## Figures and Tables

**Table 1:** Composition of artificial blended textile samples in weight percentage.

Sample	Cotton (%)	Wool (%)	PET (%)
**CWP_90/5/5**	90	5	5
**CWP_80/5/10**	80	5	15
**CWP_80/10/10**	80	10	10
**CWP_70/10/20**	70	10	20
**CWP_60/10/30**	60	10	30
**CWP_50/10/40**	50	10	40

## Supplementary Materials

The following are available online at http://www.mdpi.com/2073-4360/10/10/1107/s1, Figure S1: Recovery of protein and cellulose fibers building blocks after sequential treatment; Figure S2 and S3: Protease volumetric assay; Figure S4: SDS-Page of the samples after protease treatment; Figure S5: Amino groups content from wool hydrolysate supernatants; Figure S6: FT-IR spectra of the recovered PET materials; Figure S7: Weight % recovered PET.
