# Supplementary material for: Highly Selective Enzymatic Recovery of Building Blocks from Wool-Cotton-Polyester Textile Waste Blends
Source: Polymers (Basel). 2018 Oct 7;10(10):1107. doi: 10.3390/polym10101107 (PMC6403871; doi:10.3390/polym10101107)
Supplement: Supplementary file 1 [file polymers-10-01107-s001.pdf]

Enzymatic recovery of building blocks from wool-cotton-polyester textile waste from textile blends

F. Quartinello[(1)],S. Vecchiato[(2)], S. Weinberger [(2)], K. Kremenser [(2)], L. Skopek [(2], A. Pellis[(3,*)], G. M. Guebitz[(1,2)]

[(1)] *University of Natural Resources and Life Sciences Vienna, Dep. of Agrobiotechnology IFA-Tulln, Inst. of Environ. Biotech., Konrad Lorenz Strasse 20, 3430, Tulln a. d. Donau, Austria*

[(2)] *Austrian Centre of Industrial Biotechnology, Division Polymers & Enzymes, Konrad Lorenz Strasse 20, 3430, Tulln a. d. Donau, Austria*

[(3)] *University of York, Department of Chemistry, Green Chemistry Centre of Excellence, Heslington, York YO10 5DD, UK*

[*] *Corresponding author: Alessandro Pellis PhD,* alessandro.pellis@gmail.com

Keywords: Textile recycling, sustainable processes, protease, amino acids, cellulases, bioe thanol, poly(ethylene terephthalate), circular economy.

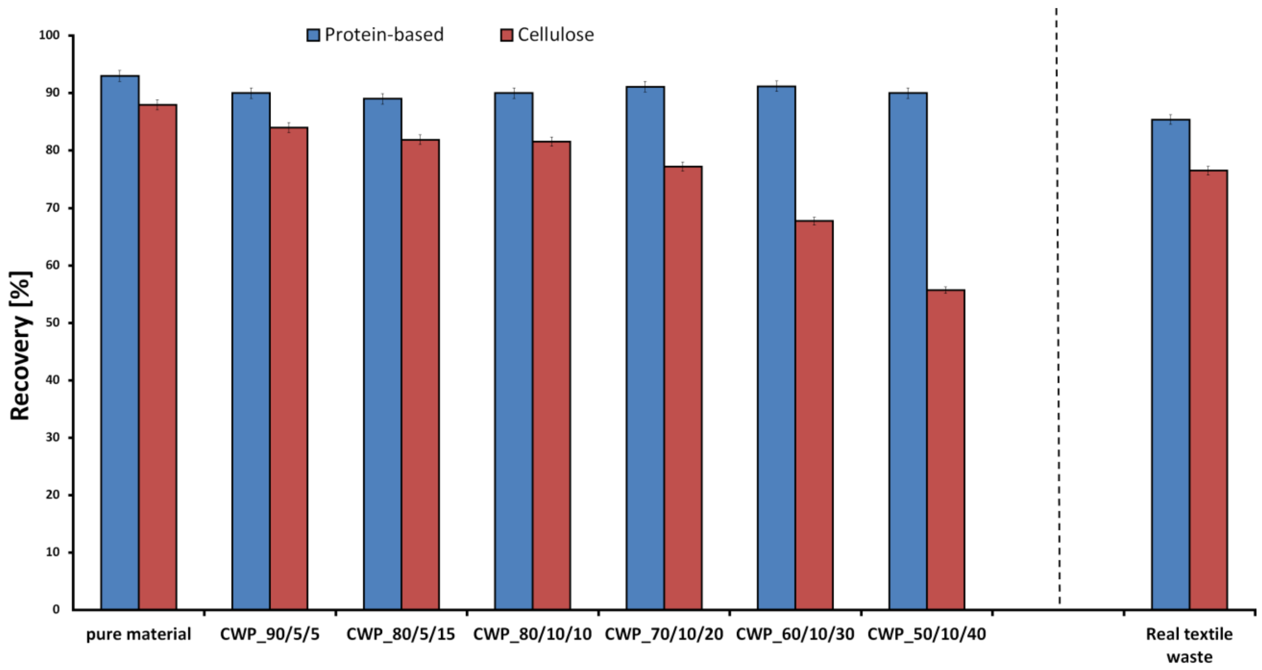

Figure S1 Recovery of protein and cellulose fibre building blocks after sequential treatment of textile waste and model mixtures with proteases and cellulases, respectively depending on cellulose (C), wool (W) and polyester (P) content. The data are given in (%) related to weight loss from the starting material (test performed in triplicates).

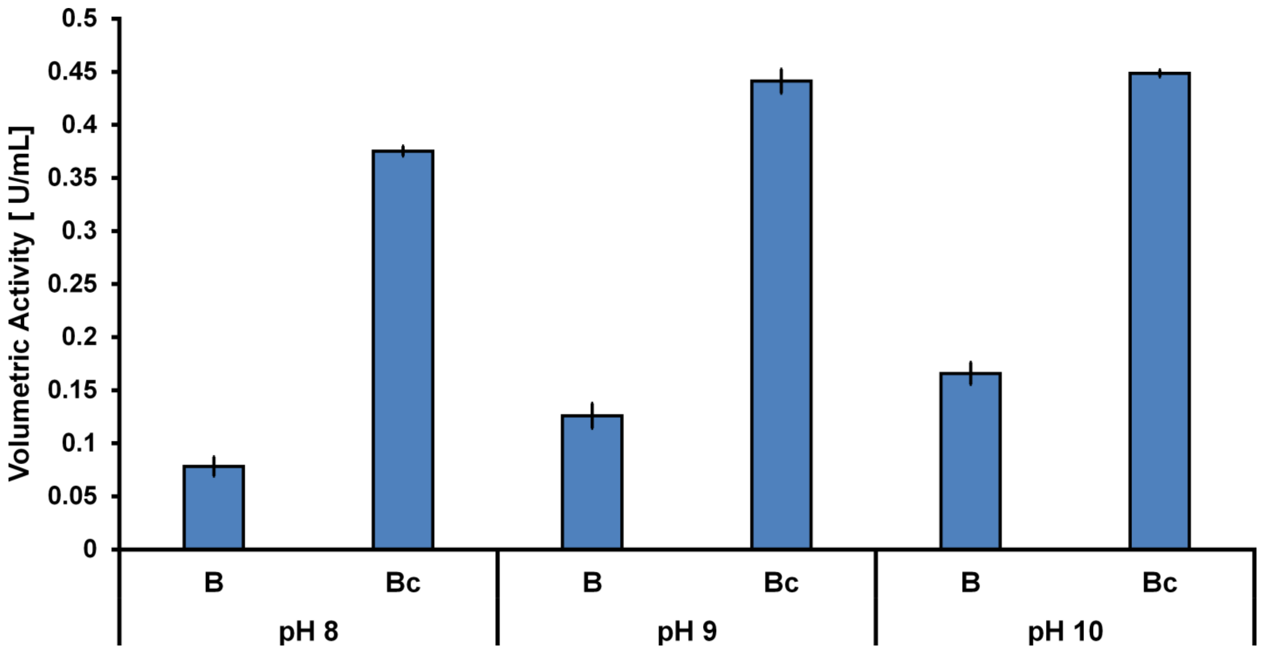

Figure S2 Volumetric activity of protease with (B) and without SDS and sodium bisulfite (B_c) at pH 8, 9 and 10 with azocasein assay. Experiments were performed in triplicate

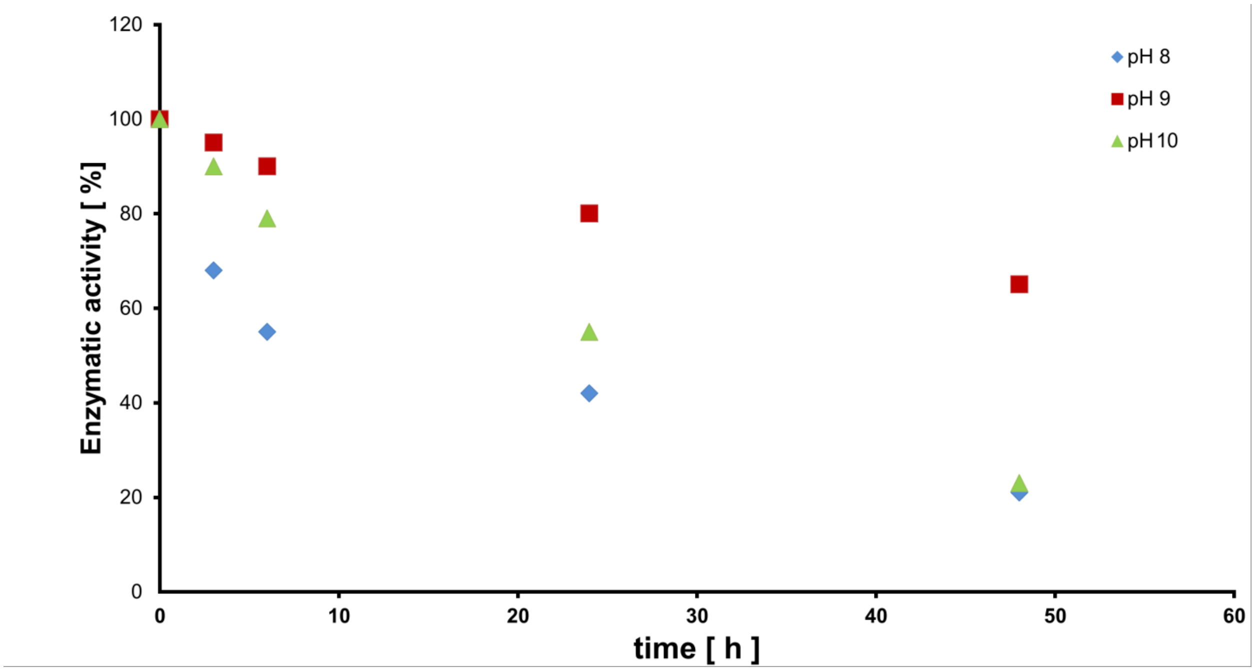

Figure S3 Volumetric activity of protease with SDS and sodium bisulfite at pH 8, 9 and 10, with azocasein assay. Experiments were performed in triplicate.

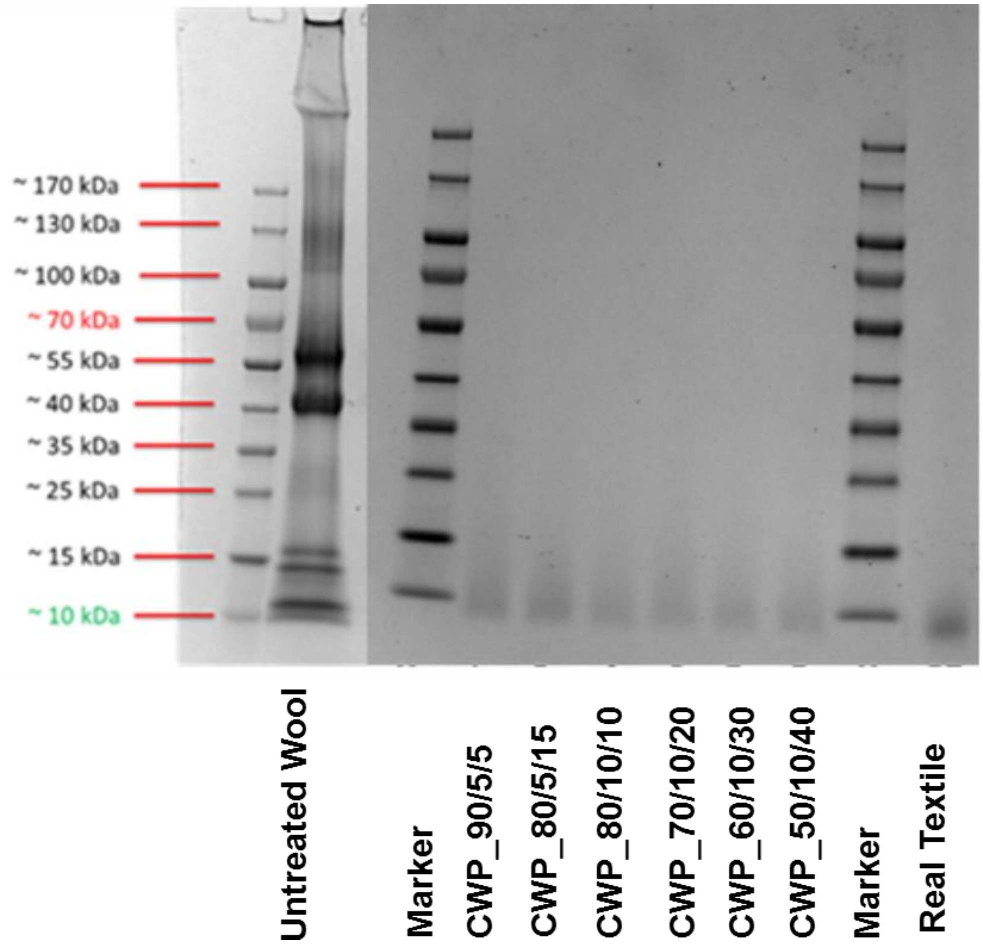

Figure S4 SDS-Page of protein bands after protease treatment.

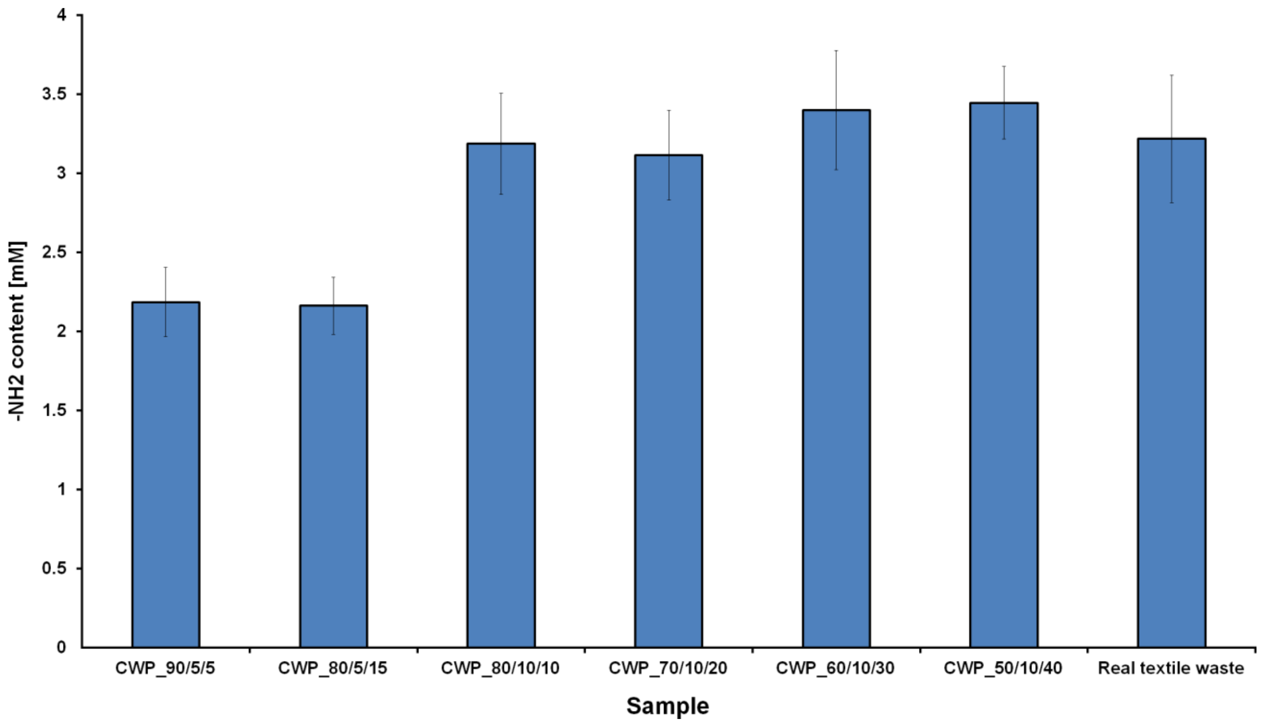

Figure S5 Amino groups content from wool hydrolysate supernatants.

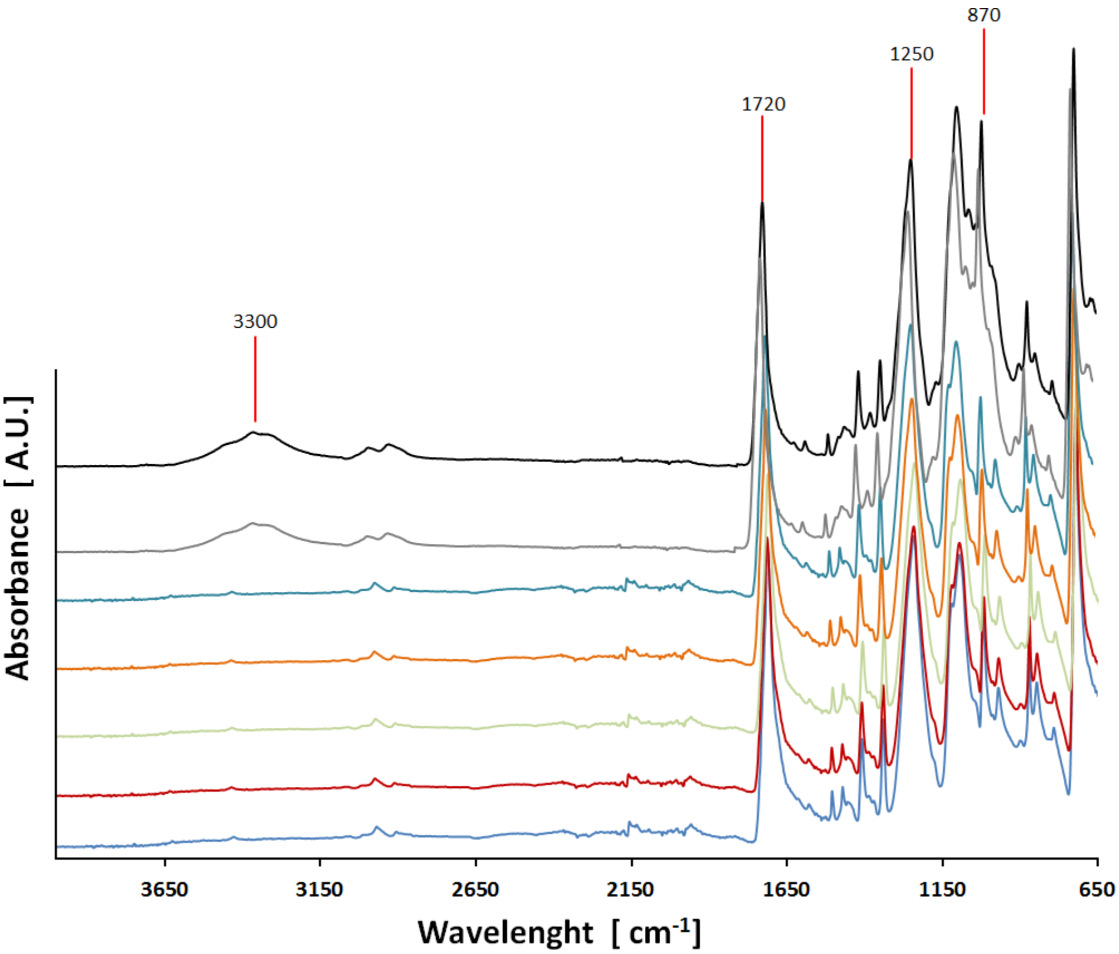

Figure S6 FT-IR spectra of: pure PET (blue line), CWP_90/5/5 (red line), CWP_80/5/15 (grey line), CWP_80/10/10 (orange line), CWP_70/10/20 (light blue line), CWP_60/10/30 (dark grey line), CWP_50/10/40 (black line). All the spectra were normalized in the area 2500-2200 cm^{-1}.

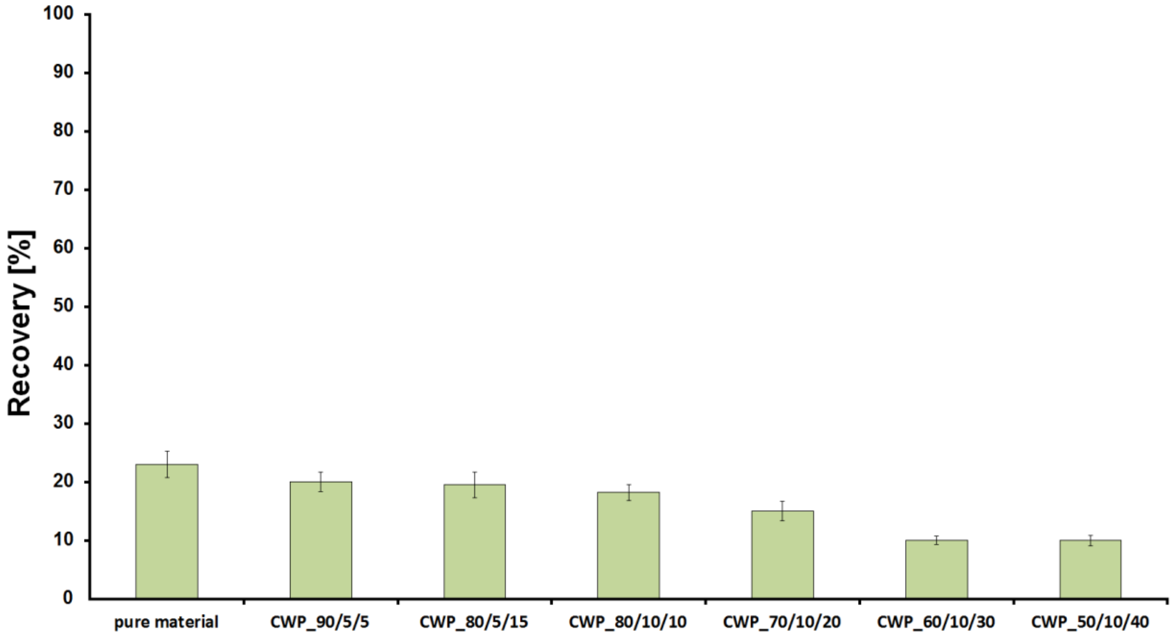

Figure S7 Recovery of PET building blocks with *Humicola insolens* cutinase
